# Supplementary material for: A subset of antibodies targeting citrullinated proteins confers protection from rheumatoid arthritis
Source: Nat Commun. 2023 Feb 8;14:691. doi: 10.1038/s41467-023-36257-x (PMC9908943; doi:10.1038/s41467-023-36257-x)
Supplement: Supplementary file 1 — Supplementary information [file 41467_2023_36257_MOESM1_ESM.pdf]

SUPPLEMENTARY INFORMATION

A subset of antibodies targeting citrullinated proteins confers protection from rheumatoid arthritis

Yibo He[1], Changrong Ge[1], Àlex Moreno-Giró[1,2], Bingze Xu[1], Christian M. Beusch[3], Katalin Sandor[4], Jie Su[5], Lei Cheng[1], Erik Lönnblom[1], Christina Lundqvist[6], Linda M. Slot[7], Dongmei Tong[1], Vilma Urbonaviciute[1], Bibo Liang[1,8], Taotao Li[1], Gonzalo Fernandez Lahore[1], Mike Aoun[1], Vivianne Malmström[9], Theo Rispens[10], Patrik Ernfors[5], Camilla I. Svensson[4], Hans Ulrich Scherer[7], René E M Toes[7], Inger Gjertsson[6], Olov Ekwall[6,11], Roman A. Zubarev[3], Rikard Holmdahl[1,8]

[1]Section for Medical Inflammation Research, Department of Medical Biochemistry and Biophysics, Karolinska Institutet, 171 77 Stockholm, Sweden

[2]Redoxis AB, 223 81 Lund, Sweden

[3]Division of Physiological Chemistry I, Department of Medical Biochemistry and Biophysics, Karolinska Institutet

[4]Department of Physiology and Pharmacology, Center for Molecular Medicine, Karolinska Institutet, 17177 Stockholm, Sweden.

[5]Division of Molecular Neurobiology, Department of Medical Biochemistry and Biophysics, Karolinska Institutet, 171 77 Stockholm, Sweden

[6]Department of Rheumatology and Inflammation Research, Institute of Medicine, Sahlgrenska Academy, University of Göteborg, Sweden

[7]Department of Rheumatology C1-R, Leiden University Medical Center, PO Box 9600, Leiden, 2300RC, The Netherlands.

[8]Center for Medical Immunopharmacology Research, Southern Medical University, Guangzhou, China

[9]Department of Medicine, Division of Rheumatology, Center for Molecular Medicine, Karolinska Institutet, Karolinska University Hospital, Stockholm, Sweden

[10]Department of Immunopathology, Sanquin Research and Landsteiner Laboratory, Academic Medical Center, University of Amsterdam, 1066 CX Amsterdam, the Netherlands

[11]Department of Pediatrics, Institute of Clinical Sciences, Sahlgrenska Academy, University of Göteborg, Sweden

Correspondence to Rikard Holmdahl, MD, Ph.D., Karolinska Institutet, Department of Medical Biochemistry and Biophysics, Medical Inflammation Research, SE-171 77 Stockholm, Sweden.

E-mail: Rikard.Holmdahl@ki.se.

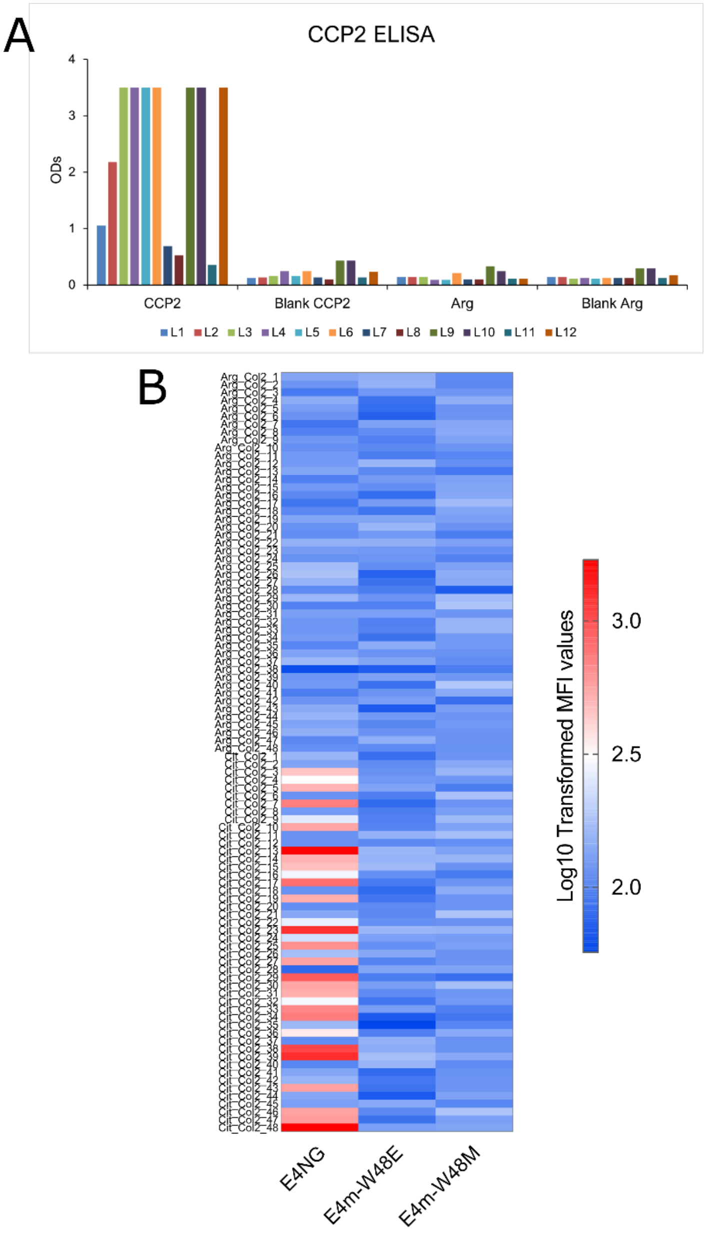

Fig.S1. Reactivity of single B cell culture or mAbs to citrullinated peptides. (A) CCP2 reactivity of indicated single B cell culture (L1-L12) from RA patients. The OD values from ELISA results are presented with an upper limit of 3.5. (B) Reactivity of E4m variants to citrullinated COL2 peptides with correspondent unmodified (arginine) peptides tested by Luminex, data are presented as Log10 transformed MFI values in heatmap.

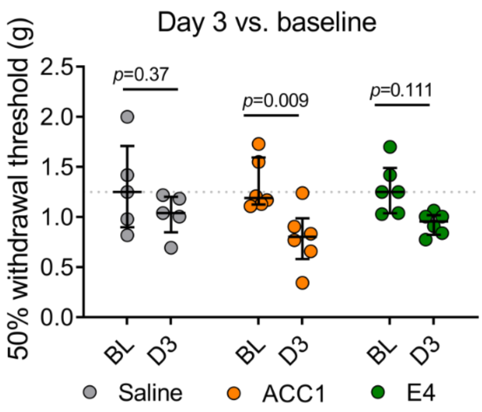

Fig.S2. Evoke pain-like behavior test on mAbs. Antibody-evoked pain-like behavior assessed by von Frey Filaments. 4 mg of E4, ACC1 or saline were intravenously injected to C57BL6/N mice on day 0 (n = 5 for saline and 6 for others). Withdrawal thresholds were measured on baseline (BL, 3 days before antibody injection) and day 3, the 50% withdrawal thresholds were calculated using the Dixon up-down method. Data are analyzed by using two-way ANOVA and presented as median with interquartile range.

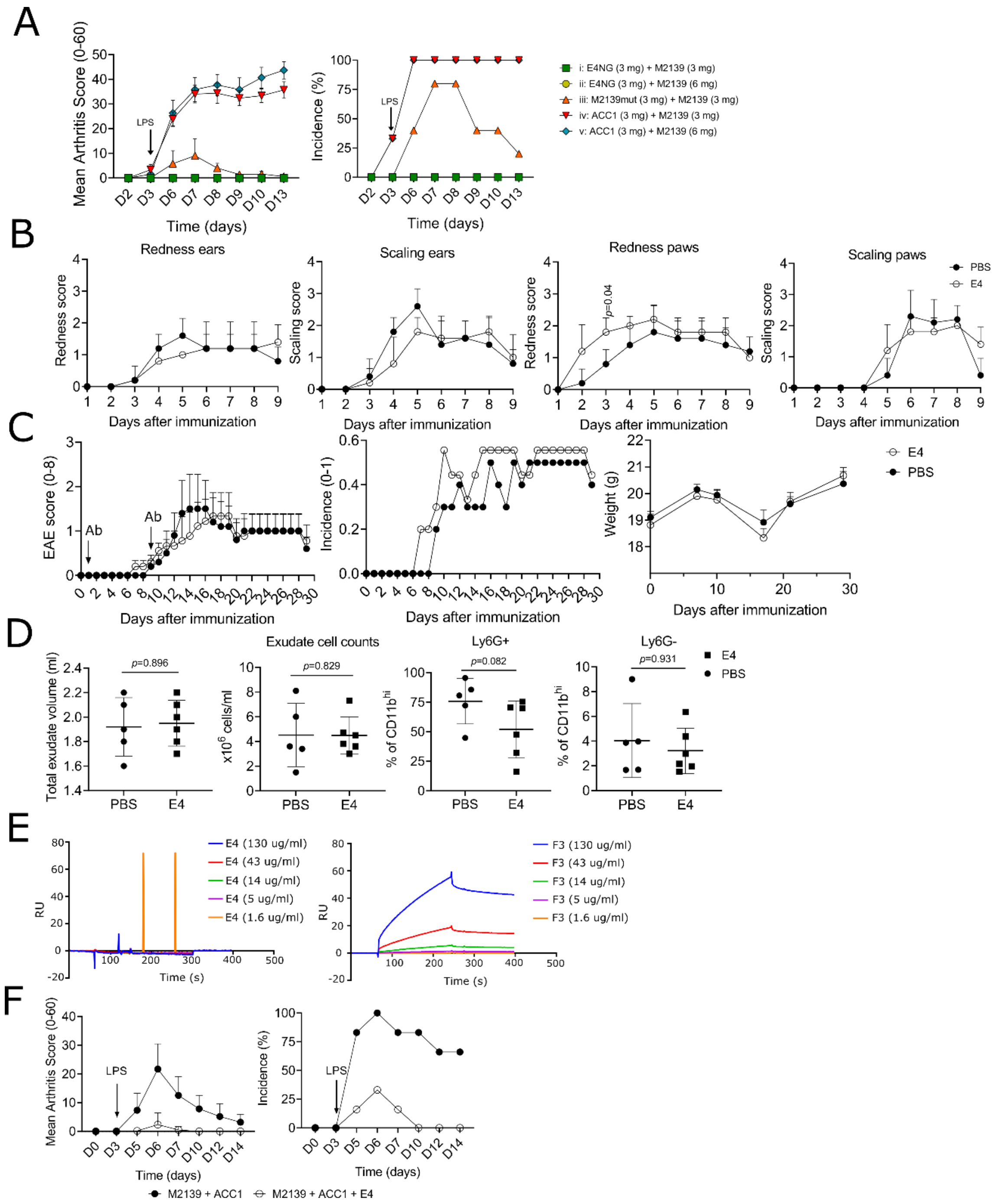

Fig.S3. Effects of E4 ACPA in different mouse models. (A) Protective effect of E4NG in CAIA. Indicated antibodies were intravenously injected to Cia9i mice on day 0 (n = 5 for group iii and 6 for others), followed by an LPS boost (25 µg) on day 3. Arthritis scores are assessed using Mann-Whitney test and presented as mean ± SEM. (B) E4 does not protect against mannan-induced psoriasis (MiP) in mice. Psoriasis in B6.Ncf1$^{-/-}$ mice was induced by intraperitoneal injection of 20 mg of mannan on day 0, together with 3 mg of E4 or PBS (n=5). Data are assessed using Mann-Whitney test and presented as mean ± SD. (C) E4 does not protect against mouse experimental autoimmune encephalomyelitis (EAE) model. 100 µl of MOG$_{33\text{-}55}$ peptide (200 µg)/CFA emulsion together with 200 ng of pertussis toxin (PTX) were injected on day 0. 1 mg of E4 (n=9) or PBS (n=10) was injected on day 1 and 9. A second dose of PTX was injected on day 2. The disease scores are assessed using Mann-Whitney test and presented as mean ± SEM. (D) E4 does not suppress cell infiltration in mouse carrageenan air pouch model. On day 0, 5 ml of air was subcutaneously injected to the back of the mice, together with 2 mg of E4 (n=6) or PBS (n=5) injected intraperitoneally. 2 ml of additional air was injected on day 3 followed by an injection of 1 ml of 2% carrageenan on day 6. The exudates were collected on day 7 and analyzed by flow cytometry for the frequencies of neutrophils (Singlets/live/CD11b^{+}/Ly6G^{+}) and monocytes (Singlets/live/CD11b^{+}/Ly6G^{-}). Data are analyzed using Mann-Whitney test (two-tailed) and presented as mean ± SD. (E) Surface plasmon resonance (SPR) sensorgram of E4 and F3 Fab fragment binding to immobilized citrullinated histone H2A peptide. Response units (RU) reflect the value of substracting H2A channel from empty channel. (F) E4 protects against CAIA in Balb/c.Ncf1^{m1j} mice. CAIA was induced by intraperitoneally injecting 2 mg of M2139 and ACC1 to Balb/c.Ncf1^{m1j} mice on day 0 (n = 6), followed by LPS boost on day 3. Data were analyzed using Mann-Whitney test (two-tailed) and presented as mean ± SD.

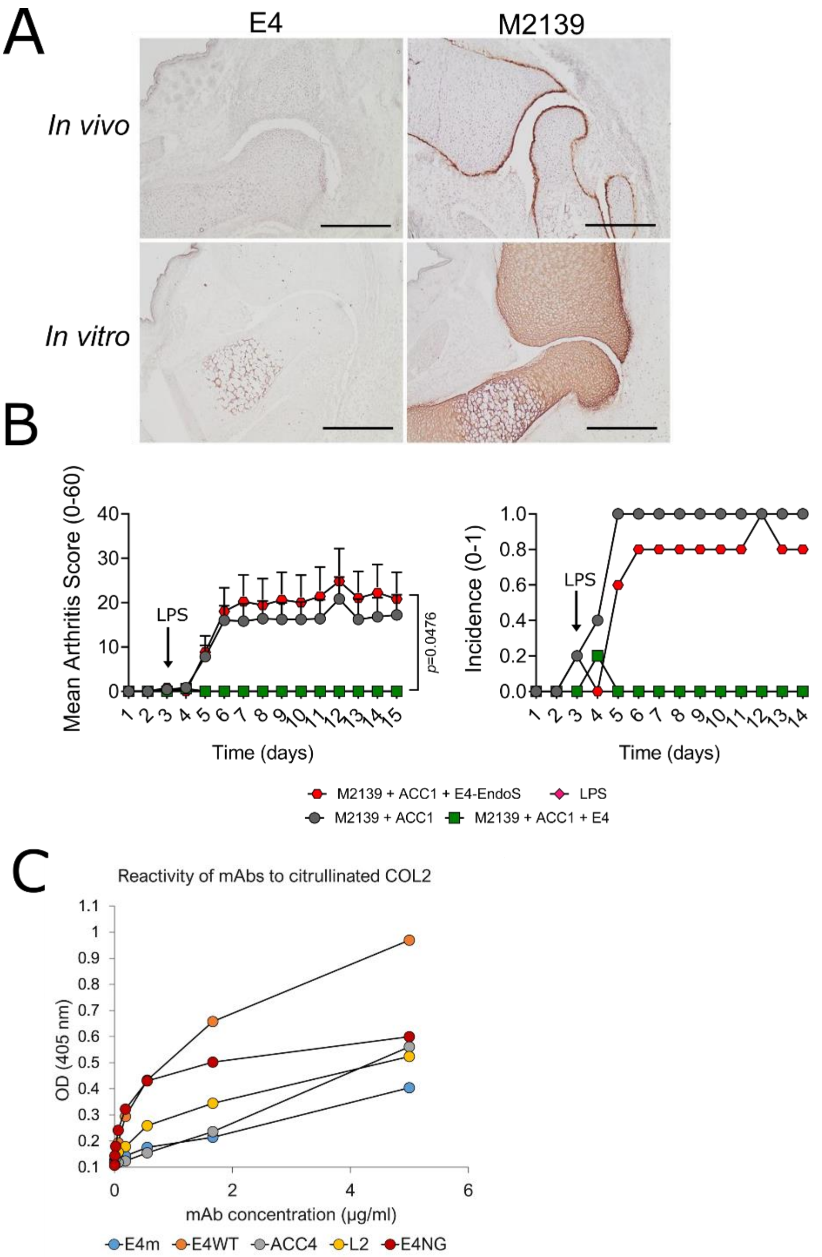

Fig.S4. Reactivity of E4 ACPA to naïve cartilage and the effect on CAIA. (A) E4 does not bind to naïve cartilage *in vivo* and *in vitro*. The knee joints and paws were taken from neonatal Cia9i mice for immunohistochemical staining, samples were examined by light microscopy (20×magnification), scale bars represent 100 µm. (B) E4 protects against CAIA in DBA/1 mice. Arthritogenic antibodies (M2139 + ACC1, 2 mg/Ab) with or without 3 mg of E4 were injected to wildtype DBA/1 mice on day 0(n = 5), followed by a boost by LPS on day 3. Arthritis scores and incidence evaluated in different time points were shown. Data are analyzed by Mann-Whitney test and presented as mean ± SEM. (C) Reactivity of ACPAs to citrullinated COL2. Recombinant bovine COL2 was citrullinated by hPAD4 with 100 mM $Ca2^{+}$ overnight (E/S ratio = 1:10), the reaction was stopped by EDTA and used for coating in 96-well plate for ELISA (5 µg/ml). The antibody binding was detected using goat anti-mouse IgG Fc secondary antibody and developed by ABTS assay, and results were shown in curves with different antibody concentrations.

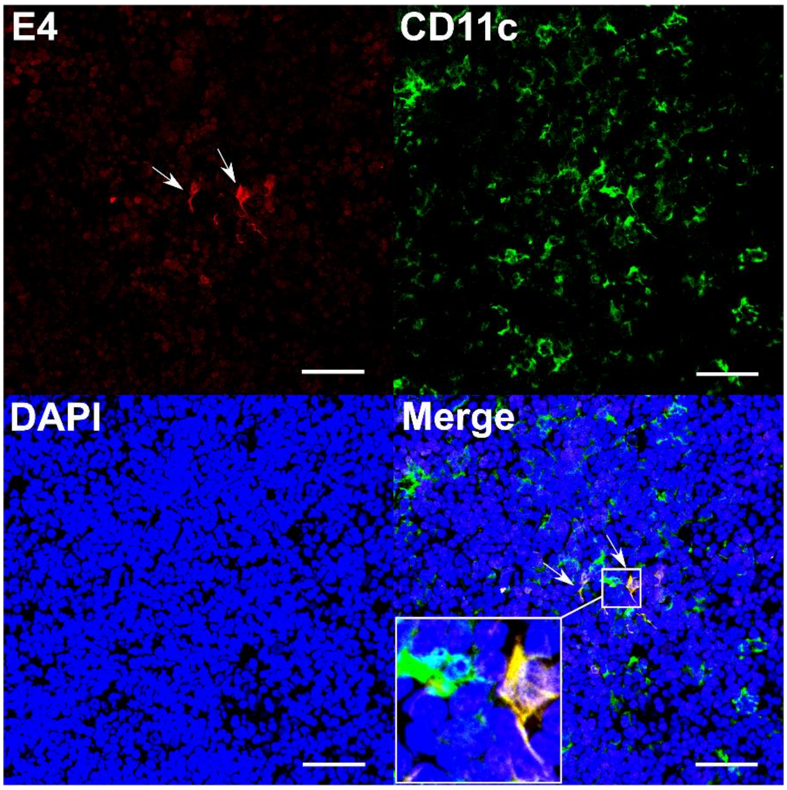

Fig.S5. Co-localization of CD11c and E4 in human thymus. CD11c, biotinylated E4 and nuclei were indicated by green, red and blue color, respectively. Images were captured by LSM700 at 10× magnification and scale bars represent 50 µm.

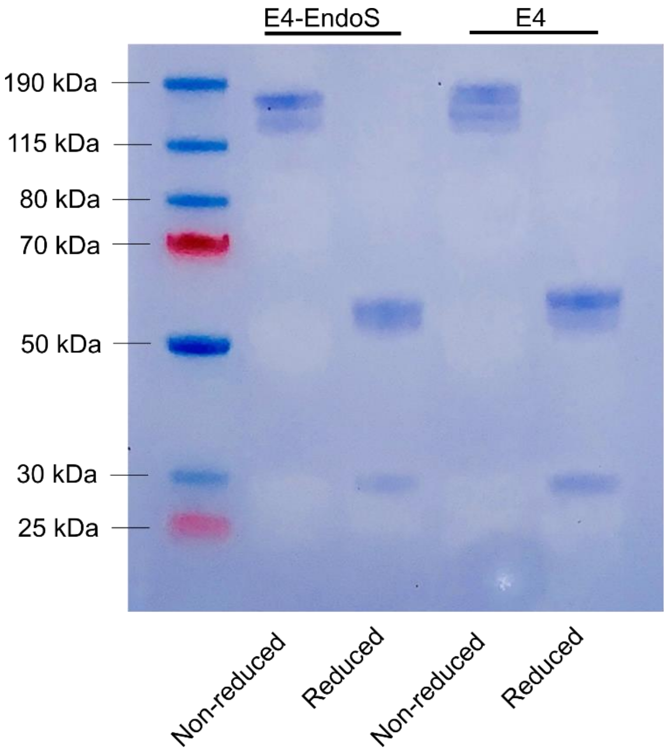

Fig.S6. Cleavage of Fc N-glycan of E4 ACPA. Coomassie blue staining on SDS-PAGE gel. The E4 and E4 antibody treated with endoglycosidase S (E4-EndoS) were shown in either reduced or non-reduced condition. A clear cleavage of the Fc-glycans could be interpreted by a reduced size of heavy chain with Endo S treatment vs. w/o treatment.

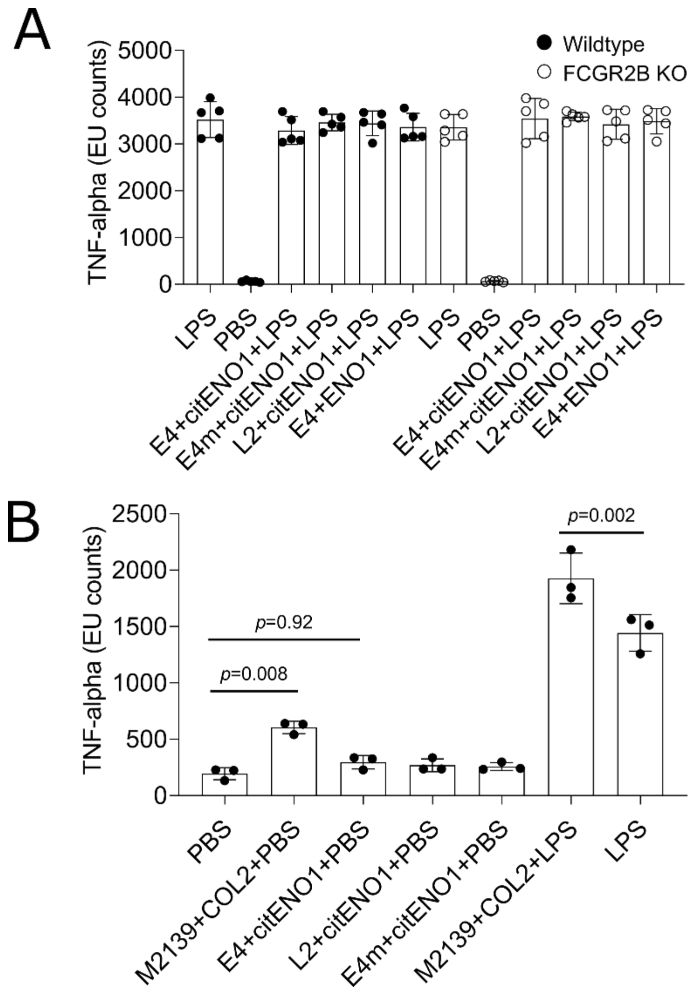

Fig.S7. Effect of immune complex treatment on TNF-alpha secretion by macrophages. (A) The indicated antibodies with citENO1 were prepared as indicated in relevant section, BMDMs from WT or FCGR2B KO mice were differentiated for 10 days before IC (10 µg/ml) or LPS (500 ng/ml) treatment overnight. On day 11, supernatants were collected and concentrated for TNF-alpha sandwich ELISA, developed by streptavidin using the Europium assay. (B) Pathogenic immune complex induces/enhances TNF-alpha secretion by macrophages. Experiment was performed similarly as above using WT mouse BMDMs. As a control the naïve or LPS-stimulated BMDMs were treated with M2139 antibody in complex with COL2. All data are analyzed by one-way ANOVA and presented as mean ± SD.

SUPPLEMENTARY TABLES

Table S1. Animal strains used in this study.

Mouse strain	Application in this study
DBA/1	Collagen antibody-induced arthritis[1,2], glucose-6-phosphate isomerase (G6PI) induced arthritis[3]
B10.Q.Cia9i	Collagen antibody-induced arthritis
B10.Q	Macrophage differentiation, air-pouch model[4]
B10Q.FCGR2B$^{-/-}$ (FCGR2B KO)	Collagen antibody-induced arthritis, macrophage differentiation
Balb/c	Macrophage differentiation, evoked pain-like behavior test[5]*
Balb/c.Ncf1^{m1j}	Collagen antibody-induced arthritis
C57BL6/N	Experimental autoimmune encephalomyelitis[4], evoked pain-like behavior test
B10.RIII	Evoked pain-like behavior test[6]
B10Q.Nfc1$^{-/-}$	Mannan-induced psoriasis[7]

*Female mice were used.

Table S2. Parameter settings for mass spectrometry analysis.

Parameter	Macrophage proteins	Human synovial fluid proteins
Gradient	80 min (4%-26% in 55 min, to 40% in 10 min, to 95% in 3 min, 95% for 4 min, to 4% in 1 min, hold for 8 min)	110 min (4%-26% in 80 min, to 32% in 10 min, to 95% in 5 min, 95% for 5 min, to 4% in 1 min, hold for 9 min)
Scan cycle (s)	2	3
MS1 resolution (Th)	120`000	120`000
MS1 scan range (Th)	350-1700	350-1700
Injection time (ms)	50	50
MS1 AGC	1`000`000	1`000`000
Included charge states	2+ to 7+	2+ to 7+
Exclusion time (s)	45	45
MS2 Isolation window (Th)	1.6	1.6
MS2 NCE (%)	28	28
MS2 resolution	30`000	30`000
Injection time (ms)	54	54
MS2 AGC	125`000	125`000

REFERENCES

1. Nandakumar, K. S. & Holmdahl, R. Efficient promotion of collagen antibody induced arthritis (CAIA) using four monoclonal antibodies specific for the major epitopes recognized in both collagen induced arthritis and rheumatoid arthritis. *J. Immunol. Methods* **304**, 126–136 (2005).

2. Khachigian, L. M. Collagen antibody-induced arthritis. *Nat. Protoc.* **1**, 2512–2516 (2006).

3. Bockermann, R., Schubert, D., Kamradt, T. & Holmdahl, R. Induction of a B-cell-dependent chronic arthritis with glucose-6-phosphate isomerase. *Arthritis Res. Ther.* **7**, R1316-24 (2005).

4. Duarte, D. B., Vasko, M. R. & Fehrenbacher, J. C. Models of Inflammation: Carrageenan Air Pouch. *Curr. Protoc. Pharmacol.* **56**, 5.6.1-5.6.8 (2012).

5. Mogil, J. S. *et al.* Heritability of nociception I: Responses of 11 inbred mouse strains on 12 measures of nociception. *Pain* **80**, 67–82 (1999).

6. Bersellini Farinotti, A. *et al.* Cartilage-binding antibodies induce pain through immune complex–mediated activation of neurons. *J. Exp. Med.* **216**, 1904–1924 (2019).

7. Khmaladze, I. *et al.* Mannan induces ROS-regulated, IL-17A–dependent psoriasis arthritis-like disease in mice. *Proc. Natl. Acad. Sci.* **111**, (2014).
