# Supplementary material for: A subset of antibodies targeting citrullinated proteins confers protection from rheumatoid arthritis
Source: Nat Commun. 2023 Feb 8;14:691. doi: 10.1038/s41467-023-36257-x (PMC9908943; doi:10.1038/s41467-023-36257-x)
Supplement: Supplementary file 3 — Reporting Summary [file 41467_2023_36257_MOESM3_ESM.pdf]

nature portfolio

Corresponding author(s): Rikard Holmdahl

Last updated by author(s): Jan 17, 2023

Reporting Summary

Nature Portfolio wishes to improve the reproducibility of the work that we publish. This form provides structure for consistency and transparency in reporting. For further information on Nature Portfolio policies, see our Editorial Policies and the Editorial Policy Checklist.

Statistics

For all statistical analyses, confirm that the following items are present in the figure legend, table legend, main text, or Methods section.

n/a	Confirmed	
☐	☒	The exact sample size (*n*) for each experimental group/condition, given as a discrete number and unit of measurement
☐	☒	A statement on whether measurements were taken from distinct samples or whether the same sample was measured repeatedly
☐	☒	The statistical test(s) used AND whether they are one- or two-sided<br>*Only common tests should be described solely by name; describe more complex techniques in the Methods section.*
☒	☐	A description of all covariates tested
☒	☐	A description of any assumptions or corrections, such as tests of normality and adjustment for multiple comparisons
☐	☒	A full description of the statistical parameters including central tendency (e.g. means) or other basic estimates (e.g. regression coefficient) AND variation (e.g. standard deviation) or associated estimates of uncertainty (e.g. confidence intervals)
☐	☒	For null hypothesis testing, the test statistic (e.g. *F*, *t*, *r*) with confidence intervals, effect sizes, degrees of freedom and *P* value noted<br>*Give P values as exact values whenever suitable.*
☒	☐	For Bayesian analysis, information on the choice of priors and Markov chain Monte Carlo settings
☒	☐	For hierarchical and complex designs, identification of the appropriate level for tests and full reporting of outcomes
☒	☐	Estimates of effect sizes (e.g. Cohen's *d*, Pearson's *r*), indicating how they were calculated

Our web collection on statistics for biologists contains articles on many of the points above.

Software and code

Policy information about availability of computer code

Data collection	Bio-Plex Manager 6.2; Biacore 2000 software; ZEN 3.0 (blue edition); Attune NxT Software v3.1; BD FACSDiva v7.0; Thermo Xcalibur 4.0.
Data analysis	Fiji (ImageJ 1.52h); Flowjo v10.8.1; Graphpad Prism v9.4.0; R v4.1.1; Biorender; MaxQuant v1.6.2.3; Microsoft Excel.

For manuscripts utilizing custom algorithms or software that are central to the research but not yet described in published literature, software must be made available to editors and reviewers. We strongly encourage code deposition in a community repository (e.g. GitHub). See the Nature Portfolio guidelines for submitting code & software for further information.

Data

Policy information about availability of data

All manuscripts must include a data availability statement. This statement should provide the following information, where applicable:

- Accession codes, unique identifiers, or web links for publicly available datasets
- A description of any restrictions on data availability
- For clinical datasets or third party data, please ensure that the statement adheres to our policy

All The mass spectrometry-based proteomics data have been deposited to ProteomeXchange77 Consortium via the PRIDE partner repository with data set identifier PXD035177 (https://www.ebi.ac.uk/pride/archive/projects/PXD035177) and PXD035180 (https://www.ebi.ac.uk/pride/archive/projects/PXD035180) for the macrophage and human synovium fluids samples, respectively. All other data are included in the Supplemental Information or available from the authors upon reasonable requests, as are unique reagents used in this AArticle. The raw numbers for charts and graphs are available in the Source Data file whenever possible.

Human research participants

Policy information about studies involving human research participants and Sex and Gender in Research.

Reporting on sex and gender	The experiments using human samples in the present study focus on tissue staining or molecular analysis with small sample size (n<5), the samples were randomly selected based only on clinical criteria, the findings therefore do not apply to only one sex or gender.
Population characteristics	RA patients visiting LUMC with anti-CCP2 positivity, none of them was treated with B-cell depleting therapies or biologicals; RA patients (female) with anti-CEP1 positivity at clinical visits due to a need for joint effusions, with age range 27-47y and disease duration 1-16y; The cartilage explant samples originate from patients with fractured femur head or RA patients; all RA patients fulfilled the EULAR/ACR criteria for the classification of RA. The human thymic tissues were obtained from two children (age 64d old female and 153d old male), diagnosed with ventricular septal defect (VSD), and underwent corrective cardiac surgery, further genetic conditions not applicable. Buffy coats from healthy donors were obtained from Karolinska University Hospital (Solna, Stockholm). Please refer to the materials & methods section in the manuscript for more details.
Recruitment	Participants were recruited in the clinic in voluntary basis. All participants gave written informed consent priorly. Samples were randomly selected based on the experimental purpose without bias.
Ethics oversight	Local ethics committee of the LUMC, The Netherlands; regional ethics committee at the University of Gothenburg, Sweden; regional ethics committee at Karolinska Institutet, Sweden.

Note that full information on the approval of the study protocol must also be provided in the manuscript.

Field-specific reporting

Please select the one below that is the best fit for your research. If you are not sure, read the appropriate sections before making your selection.

☒ Life sciences ☐ Behavioural & social sciences ☐ Ecological, evolutionary & environmental sciences

For a reference copy of the document with all sections, see nature.com/documents/nr-reporting-summary-flat.pdf

Life sciences study design

All studies must disclose on these points even when the disclosure is negative.

Sample size	Depending on the experimental design and model, for example, in CAIA model, n=4 or 5 is the typical minimal replicate number, and in EAE model, n=10 is the typical minimal replicate number. In experiments using human samples with multiple groups, n=3 is the minimal replicate number for statistical analysis and in cell experiments with multiple groups, at least three replicates were used, as we deemed this to be sufficient based on the design of the various controls included and low observed variability between samples. More details are specified in each experimental design in the manuscript.
Data exclusions	No data exclusions in the present study.
Replication	All results were successfully replicated, more details are provided in the present manuscript text and figure legends. Experiments were repeated either exact, or similar setup.
Randomization	All samples were randomly allocated into experimental groups. Mice were kept in mixed cages to avoid cage effect.
Blinding	Animal disease model experiments were scored in a blinded manner. In vitro experiments were not blinded, as readout measurements were automated and purely quantitative, eliminating any subjective bias. As an occasion, mannual counting of osteoclast number was performed in a blinded manner.

Reporting for specific materials, systems and methods

We require information from authors about some types of materials, experimental systems and methods used in many studies. Here, indicate whether each material, system or method listed is relevant to your study. If you are not sure if a list item applies to your research, read the appropriate section before selecting a response.

Materials & experimental systems

n/a	Involved in the study	
☐	☒	Antibodies
☐	☒	Eukaryotic cell lines
☒	☐	Palaeontology and archaeology
☐	☒	Animals and other organisms
☒	☐	Clinical data
☒	☐	Dual use research of concern

Methods

n/a	Involved in the study	
☒	☐	ChIP-seq
☐	☒	Flow cytometry
☒	☐	MRI-based neuroimaging

Antibodies

Antibodies used	Chimeric monoclonal antibodies derived from RA patients and monoclonal antibodies developed in-house (detailed in Table 1 in the manuscript): E4, L1-L12, E4NG, E4m, ACC1, ACC4, M2139, E4 hIgG1 and M2139 hIgG1. These mAbs are produced in-house, therefore the commercial information is not applicable.<br><br>Commercial antibodies used in this study:<br>CD38-FITC (102705, Biolegend), CD11b-PE (557397, BD Biosciences), F4/80-PerCP-Cy5.5 (45-4801-82, Thermo Fisher Scientific), CD11b-FITC (553310, BD Biosciences), F4/80-PerCP-Cy5.5 (45-4801-82, Thermo Fisher Scientific), Ly6C-APC (128016, Biolegend), Ly6G-PE (127608, Biolegend), CD11b-PB(101224, Biolegend), CD11c-PE-Cy7 (558079, BD Biosciences), Ly6G-PE (127608, Biolegend), Ly6C-BV605 (128035, Biolegend), MHCII-FITC(553623, BD Biosciences), CD45-AF700 (103128, Biolegend), HRP-conjugated rabbit-anti-human IgG secondary antibody (P0214, DAKO), IL-10 antibody (551215, BD Biosciences), TNF-alpha antibody (559064, BD Biosciences), biotinylated IL-10 antibody (554423, BD Biosciences), biotinylated TNF-alpha antibody (558415, BD Biosciences), anti-human ENO1 antibody (PA130493, Thermo Fisher Scientific), anti-CD16/32 antibody (553141, BD Biosciences), anti-mouse IgG(H+L)-CF™488A antibody (SAB4600388, Sigma-Aldrich), anti-mouse IgG1-Alexa Fluor™ 488 antibody (A21121, Thermo Fisher Scientific), anti-human IL-10 antibody (506802, Biolegend), anti-human IL-10 antibody, biotinylated (501502, Biolegend), goat anti-mouse IgG (H+L) secondary antibody conjugated with HRP (115-035-003, Jackson ImmunoResearch).<br><br>Dilution for the used antibodies are specified in the manuscript where applicable.
Validation	All antibodies were purified by affinity chromatography, endotoxin removal, dialysis/buffer exchange and validated by specificity tests either through ELISA, IHC or Western Blot etc., detailed information could be referred to peer-reviewed publications indicated in the manuscript, the present data in the manuscript, or by the manufacturers' product page.

Eukaryotic cell lines

Policy information about cell lines and Sex and Gender in Research

Cell line source(s)	ATDC5 (99072806, Merck): derived from mouse 129 teratocarcinoma AT805; Expi293F (A14527, Thermo Fisher Scientific): derived from 293 cell line.
Authentication	For ATDC5, authentication was carried out by European Collection of Cell Cultures (ECACC), operated by Public Health England. Cells were analyzed and tested following the criteria: 1) no microbial growth vs. positive control; 2) cell count (>2x10^6 cells/amp, viability (>70%) and confluency (confluent in 2 days); 3) free of Mycoplasma contamination tested by PCR using Mycoplasma-specific primers. For Expi293F, 1) cells are demonstrated that they could be recovered as healthy logarithmically growing cells within 4 to 7 days after thawing. Viability was >90%. 2) cells are negative for Mycoplasma and 3) sterile.
Mycoplasma contamination	All cell lines were tested free of Mycoplasma contamination.
Commonly misidentified lines<br>(See ICLAC register)	No commonly misidentified lines were used.

Animals and other research organisms

Policy information about studies involving animals; ARRIVE guidelines recommended for reporting animal research, and Sex and Gender in Research

Laboratory animals	In general, male mice older than 8 weeks were used for all experiments unless specified. All mice were kept and bred in climate-controlled, specific pathogen-free (following the Felasa II protocol) environment in the facility Comparative Medicine's Annex (KM-A) of Karolinska Institutet (Stockholm, Sweden) and at the specific pathogen-free animal facility of Medicon Village (Lund, Sweden). The DBA/1 mice used in this study were purchased from Janvier Labs (#SC-DBA1-M). All animals were fed with standard rodent chow and given water ad libitum. All mice were euthanized by CO2. Strains: DBA/1, B10.Q.Cia9i, B10.Q, B10Q.FCGR2B-/- (FCGR2B KO), Balb/c, Balb/c.Ncf1m1j, C57BL6/N, B10.RIII, B10Q.Nfc1-/-.
Wild animals	No wild animals were used.

Reporting on sex	All mice used in present study were male unless specified (e.g. evoked pain-like behavior test).
Field-collected samples	No field-collected samples were used.
Ethics oversight	Stockholm Norra Djurförsöksetiska Nämnd, Stockholm, Sweden and Malmö/Lund Animal Care and Use Committee, Sweden.

Note that full information on the approval of the study protocol must also be provided in the manuscript.

Flow Cytometry

Plots

Confirm that:

- [x] The axis labels state the marker and fluorochrome used (e.g. CD4-FITC).
- [x] The axis scales are clearly visible. Include numbers along axes only for bottom left plot of group (a 'group' is an analysis of identical markers).
- [x] All plots are contour plots with outliers or pseudocolor plots.
- [x] A numerical value for number of cells or percentage (with statistics) is provided.

Methodology

Sample preparation	Mouse spleens, bone marrow-derived macrophages (BMDMs) or cells from lavage fluid collected from mouse air pouch were harvested. Samples containing RBCs were processed by ACK lysing buffer. Cells were washed by PBS thoroughly and transferred to 96-well plate. Cells were stained by Live/Dead Kit, blocked by anti-CD16/32 antibody (clone: 2.4G2), followed by staining with antibodies.<br>Please refer to materials & methods section in the manuscript for more details.
Instrument	LSR II (BD), Attune NxT (Thermo Scientific).
Software	Attune NxT Software v3.1; BD FACSDiva v7.0; Flowjo v10.8.1; Graphpad Prism v9.4.0.
Cell population abundance	No FACS sorting was performed in this study. Cell frequencies are specified in the figures with flow cytometry plots.
Gating strategy	A typical gating strategy is as follows: Lymphocytes > viability (Live/dead) > singlets > relevant markers (for example, streptavidin+ > Ly6G- > F4/80+ for antibody binding to macrophages ex vivo). Figures exemplifying gating strategy are provided in the revised manuscript.

- [x] Tick this box to confirm that a figure exemplifying the gating strategy is provided in the Supplementary Information.
